# Effects of ultraviolet B radiation and cold storage on Ram sperm morphology and physiology

**DOI:** 10.1007/s00484-025-03082-4

**Published:** 2026-02-02

**Authors:** Daniele S de S. Cestaro, Antonio Sergio Varela Junior, Anthony Valverde, Ana Paula de Souza Votto, Daza de Moraes Vaz Batista Filgueira, Marc Yeste, Carine Dahl Corcini

**Affiliations:** 1 Programa de Pós-Graduação em Ciências Fisiológicas: Fisiologia Animal Comparada, Universidade Federal do Rio Grande (FURG), Rio Grande, RS Brazil; 2 Instituto de Ciências Biológicas, Universidade Federal do Rio Grande, Rio Grande, RS Brazil; 3 ReproPel, Faculdade de Veterinária, Universidade Federal de Pelotas (UFPel), Pelotas, RS Brasil; 4 Campus Capão do Leão, Campus Universitário, S/N - CEP 96160-000 Capão do Leão, RS Brasil; 5 Escuela de Agronomía, Campus Tecnológico Local San Carlos, Instituto Tecnológico de Costa Rica, Cartago, Costa Rica; 6 Biotechnology of Animal and Human Reproduction (TechnoSperm), Institute of Food and Agricultural Technology, University of Girona, Girona, Spain; 7 Unit of Cell Biology, Department of Biology, Faculty of Sciences, University of Girona, Girona, Spain; 8 Catalan Institution for Research and Advanced Studies (ICREA), Barcelona, Spain

**Keywords:** Acrossome, Spermatozoa, Computer-assisted sperm analysis

## Abstract

Ultraviolet B radiation damages DNA increases oxidative stress and impairs fertility and survival in organisms. The spermatozoa are particularly vulnerable, experiencing altered structure, function, and fertilization capacity due to UVB exposure. The objective of the present study was to evaluate the effects of ultraviolet B radiation and cold storage on the morphology and physiology of ram spermatozoa. The samples of fresh semen from seven animals, obtained during six collections, yielded 42 ejaculates. Aliquots were diluted in Tris egg yolk and were subjected to varying doses of UVB radiation, namely no radiation (0 mJ/cm^2^), 2.199 mJ/cm^2^, 4.398 mJ/cm^2^, 6.597 mJ/cm^2^, 8.796 mJ/cm^2^, and 10.995 mJ/cm^2^. All evaluations were carried out in duplicate and immediately after UVB exposure; samples were also stored at 5 °C for analysis at 24 and 48 h. Sperm were evaluated for motility using a computer assisted sperm analysis system (CASA), apart from plasma membrane integrity, mitochondrial function, DNA integrity, and acrosomal fluorescence. While UVB exposure damaged the acrosome, the plasma membrane and DNA remained intact. Storage at 5 °C for 24 h and 48 h did not affect any of the structures studied, and even though certain aspects of sperm kinematics were affected, they retained motility. Thus, exposure to UVB resulted in lower sperm motility and structures without completely damaging the sperm.

## Introduction

Ultraviolet (UV) radiation, a segment of the solar electromagnetic spectrum, is classified into three types according to wavelength: UVA (320–400 nm), UVB (290–320 nm), and UVC (100–290 nm). While UVC is completely blocked by the atmosphere, UVB is only partially absorbed by the ozone layer, allowing a small but biologically significant portion—about 5%—to reach the Earth’s surface (Arrigo 1994). In regions such as southern Brazil, environmental levels of UVB can reach up to 558 J/cm² during periods of high solar intensity, particularly in spring and summer (Nazari et al. 2010; Corrêa 2015). Unlike UVA, which exerts indirect effects primarily through oxidative mechanisms, UVB is capable of directly damaging DNA, notably through the formation of cyclobutane pyrimidine dimers (CPDs), and promoting cellular stress responses such as apoptosis and mitochondrial dysfunction (Pfeifer et al., 2005; Lesser 2006; Vitt et al. 2020). According to Al-Sadek and Yusuf (2024), these mechanisms are particularly harmful in cells, which often lack sufficient protective structures or repair systems. Therefore, given its higher energy and potential to compromise the integrity and function of gametes, UVB was chosen as the focus of this study.

The deleterious effects of UV radiation on reproductive biology have been studied in several invertebrate models. For example, in the terrestrial earthworm *Eisenia fetida*, both UVA and UVB significantly reduce cocoon fertility and larval survival (Hamman et al. 2003), while in *Metaphire posthuma*, UV exposure increases reactive oxygen species (ROS) and promotes lipid peroxidation, compromising membrane structure and function (Misra et al. 2005). These models are relevant for understanding the cellular effects of UV-induced stress in reproductive tissues. In contrast, many marine invertebrates and fish species use external fertilization, releasing gametes into the water column where they are directly exposed to solar UV radiation. In these organisms, UVB exposure has been linked to structural damage in spermatozoa—affecting chromatin, acrosome, and motility parameters—and resulting in reduced fertilization capacity (Seaver et al. 2009; Nahon et al. 2009). Despite differences in reproductive strategies, both groups illustrate the vulnerability of reproductive cells or fertilization stages to UV radiation when directly exposed.

Although mammals such as rams (*Ovis aries*) exhibit internal fertilization, their spermatozoa can also be exposed to UV radiation under experimental or technological conditions, such as in vitro manipulation, cryopreservation, or during storage in artificial media used in reproductive biotechnologies. These conditions simulate environmental stress and allow investigation of cellular susceptibility to specific damaging agents. Ram sperm is widely used in reproductive studies due to its well-characterized physiology, economic relevance in animal production, and availability of standardized andrology protocols (Barth and Oko 1989). Furthermore, spermatozoa serve as a sensitive model for studying oxidative damage, mitochondrial dysfunction, and DNA fragmentation, due to their high mitochondrial content, limited cytoplasmic antioxidant defense, and role in transmitting paternal genetic and epigenetic information (Andersson et al. 2010; Vicente-Carrillo et al. 2015).

Sperm morphology analysis is a critical component of male fertility assessment and involves identifying both normal and abnormal cell forms, with particular attention to structural defects in the head, acrosome, midpiece, and tail regions (Kondracki et al. 2020). These morphological traits are not only indicative of the spermatogenic process but also have direct implications for fertilization outcomes. Abnormalities are commonly categorized as primary or secondary, depending on whether they arise during spermatogenesis or post-ejaculation, and as compensable or uncompensable. While compensable defects typically affect motility or viability—limiting the sperm’s ability to reach or penetrate the oocyte—uncompensable defects are more likely to interfere with fertilization or early embryonic development (Banaszewska and Andraszek 2021). In addition, morphological defects often correlate with other semen parameters, such as motility (L77Castellini et al., 2024; Ramasamy et al. 2015), ejaculate volume (Górski et al. 2017), and sperm concentration (Kondracki et al. 2020). Ultimately, altered sperm morphology can compromise overall fertility and influence the quality of embryo development (García-Vázquez et al. 2016).

Therefore, the objective of this study was to evaluate the effects of UVB radiation on ram spermatozoa, specifically assessing swimming patterns, plasma membrane integrity, mitochondrial function, and chromatin and acrosomal integrity. Additionally, the study investigated the impact of cold storage (5 °C for 24 to 48 h), a condition commonly used in reproductive biotechnology protocols, on UVB-exposed samples.

## Materials and methods

### Ethical approval

This study was approved by the Institutional Review Board following ethical principles and by the Scientific Ethics Committee at the University Federal of Pelotas, number 1946/2016.

Unless otherwise mentioned, all chemicals used in this study were obtained from the Sigma Chemical Company (St. Louis, MO, USA).

### Animals

Seven sexually mature and clinically healthy rams (*Ovis aries*) of mixed breed (SRD), housed in the premises of the Federal University of Pelotas (UFPel) with identical conditions of handling and feeding, were used in this study. The seven animals were subjected to six rounds of semen collection by the artificial vagina in the presence of female (Evans and Maxwell 1987), totaling 42 ejaculates.

Only ejaculates that, after collection, showing total motility greater ≥ 70% and sperm vigor ≥ 3 (range 0–5; CBRA, 2013) were used.

### Extenders

The extender used was Tris-egg yolk at pH 6.9 and 360 mOsm (Evans and Maxwell 1987) and the ejaculates were diluted at a ratio of 1:1 (v/v) upon collection for maintenance of the sperm. The collected and diluted material was taken to the laboratory for immediate analysis of motility and sperm vigor. In the samples that matched minimum established standards, a final dilution was performed to achieve a concentration of 4 × 10^7^ viable sperm/mL; these samples were then exposed to radiation and evaluated in triplicate. After UVB exposure, samples were stored refrigerated at 5 °C in a conditioning box (Koolmate, Minitube, Germany) for a period of 24–48 h.

### UVB irradiation protocol and treatments

To simulate controlled UVB exposure, semen samples (1 mL) from each ram were distributed into 24-well culture plates, with each row corresponding to an individual animal and each column representing a specific exposure time. The exposure was performed only once, in a closed chamber with matte black-painted walls to ensure that the UVB lamp was the sole light source, eliminating any external light interference or reflection. Radiation was delivered using a UVB lamp (VL-115 C, 30 W, peak emission at 312 nm; Vilber Lourmat, Marne Lavallée, France), positioned at a fixed distance from the samples. Irradiance was monitored with a calibrated ILT1400 radiometer (International Light Technologies), and the intensity measured at the sample surface was 73.3 µW/cm². Samples were exposed to UVB radiation for 0, 30, 60, 90, 120, or 150 s, corresponding to doses of 0; 2.199; 4,398; 6.597; 8.796; and 10.995 mJ/cm², respectively (Amaral et al. 2013). After exposure, the samples were stored at 5 °C for up to 48 h to assess immediate and delayed alterations induced by UVB radiation. Although these doses represent only a fraction of the maximum environmental UVB levels reported in southern Brazil (approximately 558 J/cm²; Nazari et al. 2010; Corrêa 2015), they were intentionally selected to avoid acute cytotoxicity and to detect sublethal structural damage to sperm cells, simulating realistic scenarios of accidental or improper light exposure during handling.

2.199 4.398 6.5978.79610.995.

### Sperm in vitro evalutation

Sperm were evaluated at 0, 24, and 48 h after exposure to radiation. All samples were warmed prior to the analysis in a water bath for 10 min at 37 °C, and 200 cells per sample were counted and analyzed, except for sperm motility.

### Sperm motility and kinematics

Sperm motility was tested using the Sperm Class Analyser^®^ program (Microptic S.L. version 3.2.0 – CASA), and the parameters analyzed were amplitude of the lateral displacement of the head (ALH, µm), beat-cross frequency (BCF, Hz), average distance of trajectory (DAP, µm), curvilinear distance (DCL, µm), progressive linear distance (DSL, µm), linearity (LIN, %), righteousness (STR, %), average path velocity (VAP, µm·s^− 1^), curvilinear velocity (VCL, µm·s^− 1^), straight line velocity (VSL, µm·s^− 1^), oscillation (WOB, %), and total and progressive motility. Each sample was analyzed after incubation at 37 °C for 10 min and in six fields in a pre-heated blade. CASA (Computer Assisted Sperm Analysis) is an automated system that provides accurate, precise, and meaningful information on individual sperm cell movement and on subpopulations of sperm cells (Matos et al. 2008; Víquez et al. 2020; Barquero et al. 2021; Gacem et al. 2021). CASA is an enhancement tool for those with the knowledge and skill required to analyze sperm (Matos et al. 2008; Verstegen et al. 2002).

### Functionality mitochondrial

Mitochondria functionality was evaluated by the technique described by Garner et al. (1986). A 20 µL aliquot of the semen sample was incubated with a combination of fluorescent probes, 13 mM rhodamine 123 (Rh123; Cat. R8004) and 7.3 mM propidium iodide (PI) for 5 min in a dark room. Cells were evaluated at 400x magnification using an epifluorescence microscope (Olympus BX 51, INC America, São Paulo, SP). Cells that showed an intense green fluorescence in the intermediate piece had intact mitochondria and were considered functionally active while those with little or no green fluorescence were considered non-functional.

### Membrane integrity

A 20 µL aliquot of the semen samples was exposed to a combination of fluorescent probes 1 µg/mL carboxyfluorescein diacetate (CFDA; C4916–25 mg) and 50 µg/mL Propidium Iodide (PI; P4170–1 g) in a solution of formaldehyde and sodium citrate, incubated for 5 min in a dark room, and evaluated at 400x magnification in an epifluorescence microscope. Cells showing only green fluorescence were considered intact, whereas cells with red and green-red fluorescence were considered damaged, as described by Harrison and Vickers (1990).

### Acrosome integrity

The semen samples were smeared on a slide, 20 µL of an PI solution added, dried, immersed in absolute ethyl alcohol (459844–1 L,) for 5 min, rinsed in PBS (Phosphate Buffer Saline), 20 µL of Conjugate lectin Arachis hypogaea – FITC (20 mg/ml) added, rinsed in deionized water, drained in a dark room, and the slides evaluated in an epifluorescence microscope at 1000x magnification. Cells were considered to have an intact acrosome only if they had no roughness, vacuoles, and emitted a green fluorescence; cells without these attributes were classified as cells with a damaged acrosome (Kawamoto et al. 1999).

### DNA integrity

DNA integrity was evaluated as described by Evenson et al. (1999). To an aliquot 10 µL of semen, 10 µL of TNE was added, after 30 s 50 mL of 1x Triton was added, and after 30 s 10 µL of Acridine Orange (2 mg/mL in deionized H2O) was added. After incubation for 5 min, the material was assessed in an epifluorescence microscope at 400x magnification. Cells were considered to have normal DNA (double-stranded) if they showed green fluorescence and cells with red or yellow fluorescence were considered to have methylated DNA (single-stranded).

### Statistical analysis

The Shapiro-Wilk normality test indicated that the data were nonparametric. The treatments were considered as independent variables, and motility time parameters such as, DAP, DCL, DSL, VAP, VCL, VSL, STR, LIN, WOB, ALH, BCF, membrane integrity, DNA integrity, and mitochondria functionality, were categorized as dependent variables. Thus, the data were normalized using an arcsin function, and the means were analyzed using an Kruskal-wallis. To allow interpretation, results were reported in their original scales. All analyses were performed using the Statistix software.

## Results

**Fig. 1:**
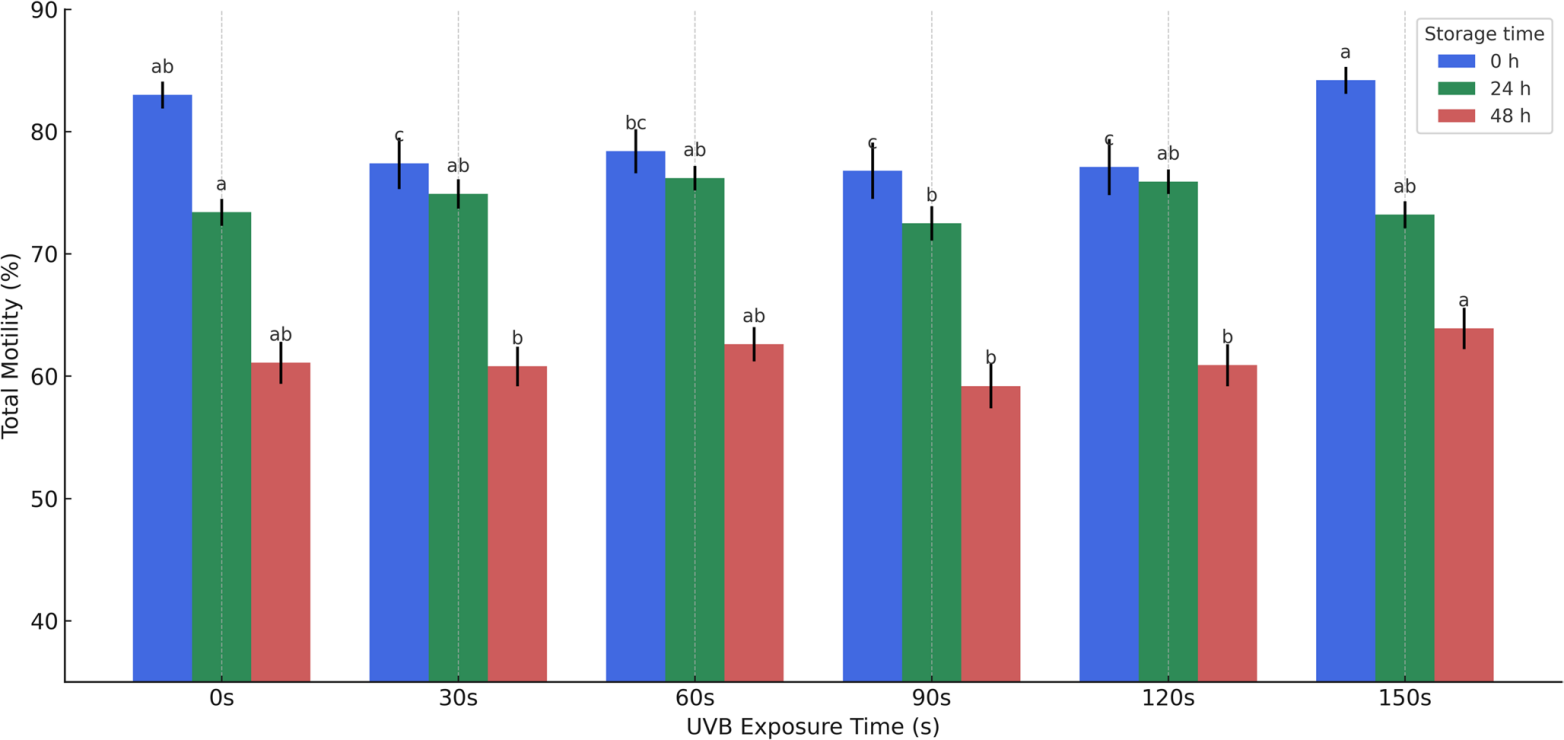
Mean values (± SEM) of total motility of ram spermatozoa immediately after UVB exposure (0 h – blue), and after 24 h (green) and 48 h (red) of cold storage at 5 °C. Sperm samples were evaluated following exposure to UVB radiation at different time intervals (0, 30, 60, 90, 120, and 150 s). Different lowercase letters above the bars indicate statistically significant differences (*p* < 0.05) within each storage timepoint, i.e., differences between exposure times at 0 h, 24 h, and 48 h, respectively. Radiation 0 s = 0; 30 s = 2.199 mJ/cm2; 60 s = 4.398 mJ/cm2; 90 s = 6.597 mJ/cm2; 120 s = 8.796 mJ/cm2; 150 s = 10.995 mJ/cm2

The results of the sperm kinematics characteristics at time zero after UVB exposure and at the other time points are presented in Table 1. Compared to control, ALH values were higher after irradiation for 150 s (10.995 mJ/cm^2^) and STR was higher after 60 s (4.398 mJ/cm^2^) of exposure. Further, total motility (Fig. 1), was reduced after exposure times of 30 s (2.199 mJ/cm^2^), 90 s (6.597 mJ/cm^2^), and 120 s (8.796 mJ/cm^2^), compared to control. Other parameters tested did not show statistically significant differences.

**Table 1:** Mean values (± SEM) of the kinematics variables of Ram sperm cells zero-time point after exposure to UVB at different times

Variables	Exposure time (s)					
	0	30	60	90	120	150
ALH	3.2 ± 0.04 ^b^	3.3 ± 0.06 ^ab^	3.2 ± 0.07 ^b^	3.2 ± 0.06 ^b^	3.3 ± 0.06 ^b^	3.5 ± 0.07 ^a^
BCF	29.5 ± 0.02 ^ab^	29.8 ± 0.2 ^a^	29.1 ± 0.3 ^b^	29.7 ± 0.3 ^ab^	29.6 ± 0.3 ^ab^	29.3 ± 0.2 ^ab^
DAP	22.9 ± 0.6	23.7 ± 0.7	22.7 ± 0.7	23.0 ± 0.7	23.5 ± 0.7	23.8 ± 0.7
DCL	37.2 ± 0.8	38.9 ± 1.0	36.7 ± 1.1	38.1 ± 1.2	38.2 ± 1.2	39.1 ± 1.1
DSL	16.1 ± 0.5	16.8 ± 0.6	16.4 ± 0.6	16.2 ± 0.6	16.7 ± 0.6	16.4 ± 0.6
LIN	0.423 ± 0.01 ^abc^	0.426 ± 0.01 ^abc^	0.438 ± 0.01 ^a^	0.420 ± 0.01 ^bc^	0.431 ± 0.01 ^ab^	0.413 ± 0.01 ^c^
STR	0.690 ± 0.01 ^bc^	0.700 ± 0.01 ^ab^	0.712 ± 0.01 ^a^	0.692 ± 0.01 ^bc^	0.698 ± 0.01 ^abc^	0.681 ± 0.01 ^c^
VAP	53.2 ± 1.2	54.3 ± 1.4	52.3 ± 1.6	52.9 ± 1.6	54.5 ± 1.5	55.8 ± 1.6
VCL	86.2 ± 1.8 ^ab^	89.7 ± 2.2 ^ab^	84.5 ± 2.4 ^b^	87.2 ± 2.5 ^ab^	88.1 ± 2.5 ^ab^	91.2 ± 2.6 ^a^
VSL	37.5 ± 1.1	39.0 ± 1.2	37.8 ± 1.3	37.2 ± 1.3	38.7 ± 1.3	38.4 ± 1.3
WOB	0.609 ± 0.01 ^ab^	0.604 ± 0.01 ^ab^	0.612 ± 0.01 ^ab^	0.601 ± 0.01 ^b^	0.614 ± 0.01 ^a^	0.605 ± 0.01 ^ab^

SEM: Standard error of the mean. Amplitude of the lateral displacement of the head (ALH, µm), beat-cross frequency (BCF, Hz), average distance of trajectory (DAP, µm), curvilinear distance (DCL, µm), progressive linear distance (DSL, µm), linearity (LIN, %), righteousness (STR, %), average path velocity (VAP, µm•s-1), curvilinear velocity (VCL, µm•s-1), straight line velocity (VSL, µm•s-1), oscillation (WOB, %). a-c Different letters in a line indicate difference statistic for Kruskal-wallis (*P* < 0.05). Radiation 0 s = 0; 30 s = 2.199 mJ/cm^2^; 60 s = 4,398 mJ/cm^2^; 90 s = 6,597 mJ/cm^2^; 120 s = 8,796 mJ/cm^2^; 150 s = 10.995 mJ/cm^2^. *N* = 42 ejaculates

Table 2 presents data on sperm kinematics after 24 h of storage at 5 °C and exposure to UVB radiation. Compared to control, the parameters LIN and STR were higher at exposure times of 90 s (6,597 mJ/cm^2^) 120 s (8,796 mJ/cm^2^), and 150 s (10.995 mJ/cm^2^), while ALH was lower at exposure times of 120 s and 150 s. The remaining parameters showed no significant changes.

Table 3 shows the results of sperm kinematics analyses at the 48 h time point. The parameters ALH, DSL, LIN, STR, and VSL showed relatively similar behavior with higher values at higher exposure times, and were unlike those observed in controls. Compared to control, WOB was reduced at 30 s exposure (2.199 mJ/cm2) but increased at 150 s exposure (10.995 mJ/cm2). Other values did not demonstrate significant changes over time or after exposure and refrigeration.

**Table 2:** Motile and kinematics variables (mean ± SEM) of Ram sperm cells 24 Hs point after exposure to UVB at different times

Variable	Exposition Time (s)					
	0	30	60	90	120	150
ALH	3.8 ± 0.05 ^a^	3.7 ± 0.1 ^a^	3.7 ± 0.1 ^a^	3.6 ± 0.1 ^ab^	3.4 ± 0.1 ^b^	3.4 ± 0.1 ^b^
BCF	29.6 ± 0.3 ^ab^	29.2 ± 0.2 ^b^	30.5 ± 0.2 ^a^	30.4 ± 0.3 ^a^	30.2 ± 0.3 ^ab^	30.5 ± 0.3 ^a^
DAP	26.4 ± 0.5	26.4 ± 0.6	27.0 ± 0.5	26.9 ± 0.5	26.3 ± 0.6	27.1 ± 0.7
DCL	48.2 ± 1.0	48.0 ± 1.1	49.5 ± 0.9	48.6 ± 0.9	46.9 ± 1.0	48.6 ± 1.1
DSL	18.1 ± 0.4	18.3 ± 0.5	19.0 ± 0.4	19.5 ± 0.4	19.2 ± 0.5	20.0 ± 0.6
LIN	0.374 ± 0.01 ^c^	0.379 ± 0.01 ^c^	0.380 ± 0,01 ^bc^	0.396 ± 0.01 ^ab^	0.405 ± 0.01 ^a^	0.403 ± 0.01 ^a^
STR	0.679 ± 0.01 ^c^	0.683 ± 0.01 ^c^	0.695 ± 0.01 bc	0.716 ± 0.01 ^ab^	0.720 ± 0.01 ^a^	0.726 ± 0.01 ^a^
VAP	61.4 ± 1.2	61.5 ± 1.4	62,5 ± 1.1	62.1 ± 1.2	60.5 ± 1.3	62.2 ± 1.5
VCL	111.8 ± 2.2	111.2 ± 2.7	114.2 ± 2.0	111.8 ± 2.1	107.6 ± 2.4	111.3 ± 2.6
VSL	42.3 ± 1.0	42.6 ± 1.1	44.1 ± 0.9	45.1 ± 1.0	44.3 ± 1.1	45.2 ± 1.3
WOB	0.548 ± 0.01 ^ab^	0.553 ± 0.01 ^ab^	0.546 ± 0.01 ^b^	0.551 ± 0.01 ^ab^	0.560 ± 0.01 ^a^	0.553 ± 0.01 ^ab^

SEM: Standard error of the mean. Amplitude of the lateral displacement of the head (ALH, µm), beat-cross frequency (BCF, Hz), average distance of trajectory (DAP, µm), curvilinear distance (DCL, µm), progressive linear distance (DSL, µm), linearity (LIN, %), righteousness (STR, %), average path velocity (VAP, µm•s^−1^), curvilinear velocity (VCL, µm•s^−1^), straight line velocity (VSL, µm•s^−1^), oscillation (WOB, %),. Different letters in a line indicate difference statistic for Kruskal-wallis (*P* < 0.05). Radiation 0 s = 0; 30 s = 2.199 mJ/cm^2^; 60 s = 4,398 mJ/cm^2^; 90 s = 6,597 mJ/cm^2^; 120 s = 8,796 mJ/cm^2^; 150 s = 10.995 mJ/cm^2^. *N* = 42 ejaculates

**Table 3:** Motile and kinematics variables (mean ± SEM) of Ram sperm cells 48 Hs point after exposure to UVB at different times

Variables	Exposition Time (s)					
	0	30	60	90	120	150
ALH	3.8 ± 0.08 ^ab^	3.9 ± 0.06 ^a^	3.6 ± 0.09 ^b^	3.8 ± 0.06 ^ab^	4.0 ± 0.04 ^a^	3.9 ± 0.05 ^a^
BCF	28.8 ± 0.3 ^cd^	29.8 ± 0.3 ^bc^	28.5 ± 0.3 ^d^	31.2 ± 0. 3 ^a^	30.5 ± 0.2 ^ab^	30.2 ± 0.2 ^ab^
DAP	27.9 ± 0.6 ^ab^	27.8 ± 0.5 ^ab^	27.2 ± 0.7 ^b^	28.2 ± 0.5 ^ab^	29.7 ± 0.5 ^a^	28.6 ± 0.5 ^ab^
DCL	52.0 ± 1.3 ^ab^	53.2 ± 1.0 ^ab^	50.3 ± 1.3 ^b^	52.4 ± 0.8 ^ab^	54.9 ± 0.8 ^a^	51.8 ± 0.9 ^ab^
DSL	18.2 ± 0.4 ^c^	18.7 ± 0.4 ^bc^	19.0 ± 0.5 ^bc^	19.6 ± 0.3 ^abc^	20.8 ± 0.4 ^a^	19.9 ± 0.4 ^ab^
LIN	0.357 ± 0.01 ^b^	0.352 ± 0.01 ^b^	0.381 ± 0.01 ^a^	0.374 ± 0.01 ^a^	0.375 ± 0.01 ^a^	0.380 ± 0.01 ^a^
STR	0.660 ± 0.01 ^c^	0.670 ± 0.0 ^bc^	0.703 ± 0.01 ^a^	0.695 ± 0.01 ^a^	0.695 ± 0.01 ^a^	0.688 ± 0.01 ^ab^
VAP	64.9 ± 1.5 ^ab^	64.4 ± 1.2 ^ab^	63.0 ± 1.7 ^b^	65.6 ± 1.1 ^ab^	68.9 ± 1.1 ^a^	66.5 ± 1.1 ^ab^
VCL	120.4 ± 2.9 ^ab^	122.4 ± 2.2 ^ab^	116.2 ± 3.1 ^b^	121.1 ± 1.9 ^ab^	126.9 ± 1.9 ^a^	119.9 ± 2.1 ^ab^
VSL	42.4 ± 0.9 ^c^	43.3 ± 0.8 ^bc^	44.0 ± 1.2 ^bc^	45.7 ± 0.8 ^abc^	48.4 ± 0.9 ^a^	46.1 ± 0.9 ^ab^
WOB	0.537 ± 0.01 ^b^	0.524 ± 0.01 ^c^	0.538 ± 0.01 ^b^	0.536 ± 0.01 ^bc^	0.539 ± 0.01 ^ab^	0.551 ± 0.01 ^a^

SEM: Standard error of the mean. Amplitude of the lateral displacement of the head (ALH, µm), beat-cross frequency (BCF, Hz), average distance of trajectory (DAP, µm), curvilinear distance (DCL, µm), progressive linear distance (DSL, µm), linearity (LIN, %), righteousness (STR, %), average path velocity (VAP, µm•s-1), curvilinear velocity (VCL, µm•s-1), straight line velocity (VSL, µm•s-1), oscillation (WOB, %),.Different letters in a line indicate difference statistic for Kruskal-wallis (*P* < 0.05). Radiation 0 s = 0; 30 s = 2.199 mJ/cm^2^; 60 s = 4,398 mJ/cm^2^; 90 s = 6,597 mJ/cm^2^; 120 s = 8,796 mJ/cm^2^; 150 s = 10.995 mJ/cm^2^. *N* = 42 ejaculates

**Fig. 2:**
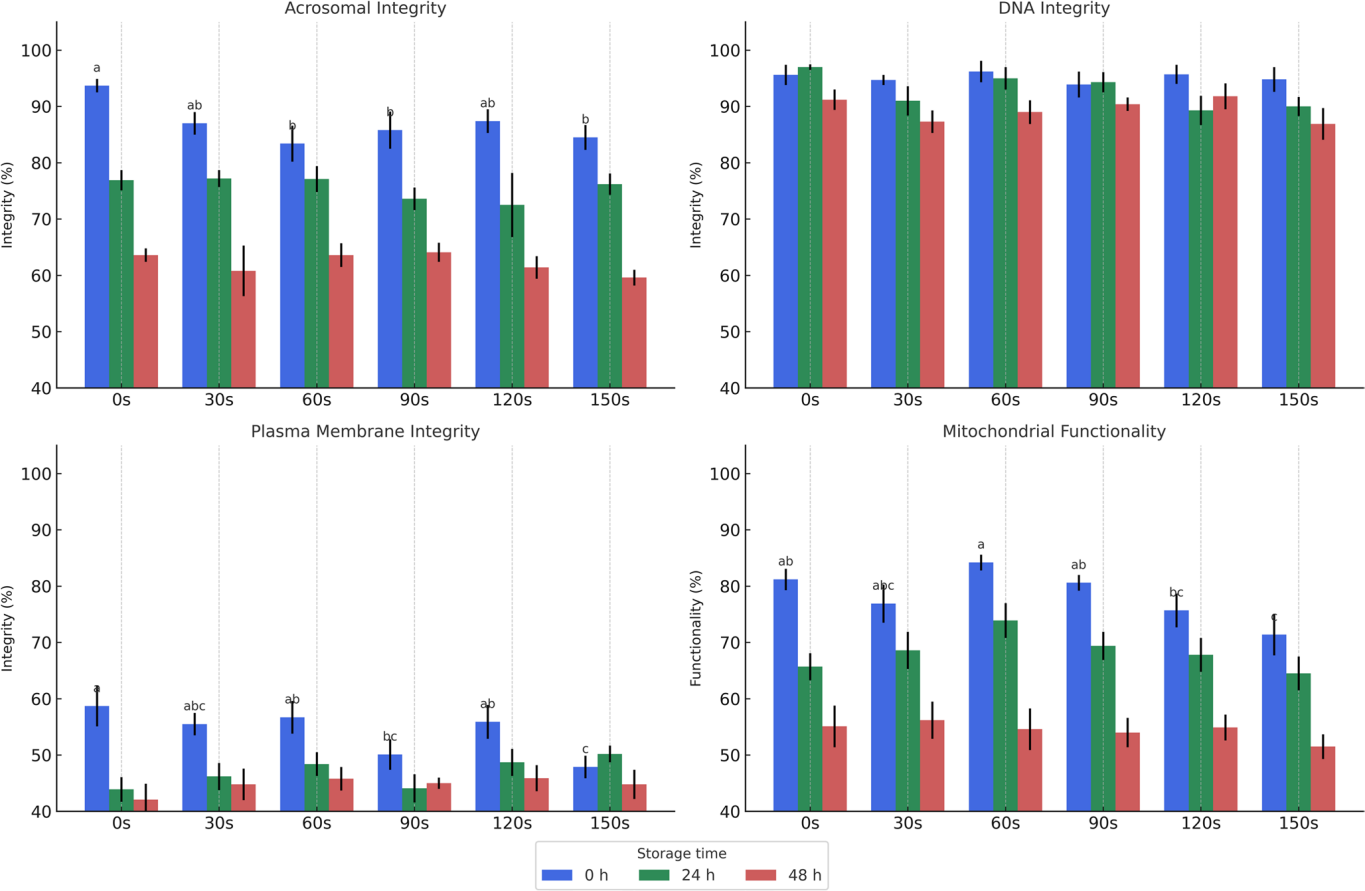
presents data on the structural integrity of ram spermatozoa after exposure to UVB radiation at different time intervals, assessed using epifluorescence microscopy. The figure includes results obtained immediately after exposure (0 h), as well as after 24 h and 48 h of cold storage at 5 °C. The analysis revealed no statistically significant DNA damage across treatments. However, UVB exposure led to a reduction in acrosomal and mitochondrial integrity, with the most pronounced damage observed after 150 s (10.995 mJ/cm²) of exposure. Moreover, the data indicate that the structural damage occurred at the moment of exposure and remained stable during the subsequent storage periods (24 h and 48 h), with no evidence of progression or recovery over time.10.995

Figure 2. Functional parameters of ram spermatozoa following exposure to ultraviolet B (UVB) radiation at different doses and evaluated immediately (0 h, blue), after 24 h (green), and after 48 h (red) of cold storage at 5 °C. Each subplot represents one functional parameter: (A) acrosomal integrity, (B) DNA integrity, (C) plasma membrane integrity, and (D) mitochondrial functionality. Bars represent mean values ± standard error of the mean (SEM) from 42 ejaculates. Different lowercase letters above the bars in the 0 h group indicate statistically significant differences among UVB exposure times (Kruskal-Wallis test, *P* < 0.05). UVB radiation doses corresponding to exposure times were as follows: 0 s = 0 mJ/cm²; 30 s = 2.199 mJ/cm²; 60 s = 4.398 mJ/cm²; 90 s = 6.597 mJ/cm²; 120 s = 8.796 mJ/cm²; 150 s = 10.995 mJ/cm².

**Discussion**.

Many studies have reported the effects of exposure to UVB radiation in reproductive cells of aquatic animals such as hedgehog-fish (Au et al. 2002; Lu and Wu 2005a, b), Mussels (Seaver et al. 2009; Cuccaro et al. 2025), and fish (Vetter et al. 1999; Browman et al. 2000; Rick et al. 2014) with a focus on environmental exposure and dose. UVB radiation has also been used in studies on human sperm (Mallidis et al. 2011; Amaral et al. 2013). This study presents an alternative approach by using spermatozoa as a highly sensitive cellular model to investigate the effects of UVB radiation on subcellular structures, with emphasis on mitochondrial function and plasma membrane integrity. Although these cells do not directly represent superficially exposed tissues such as the epidermis, their specific physiological features — including high mitochondrial activity, absence of transcriptional capacity, and limited repair mechanisms — make them valuable early indicators of oxidative stress. The use of this model advances the development of more ethical and efficient in vitro methods for environmental toxicity assessment, supporting the reduction of vertebrate animal use in experimental trials.

Mitochondria are central to successful reproduction as they provide the energy needed to beat the scourge and move along the female reproductive tract, into the oocyte, and eventually penetrate the zona pellucida (Barth and Oko 1989; O’Connell et al. 2002). Similar to results obtained by Lu and Wu (2005b), our results showed that the longer exposure time (150s) had less functional mitochondria confirming the harmful effects of UVB (Fig. 2). Quadros et al. (2016) identified mitochondria, alongside DNA, as primary cellular targets of UVB radiation. Supporting this, Hegedus et al. (2014) reported that UVB exposure induces characteristic mitochondrial damage, including potential mitochondrial uncoupling. This mitochondrial dysfunction correlates with elevated reactive oxygen species (ROS) production and a slight increase in mitochondrial membrane potential.

The cell membrane in sperm performs various processes related to cellular metabolism through selective permeability (Singer and Nicolson 1972). Our results show that sperm membrane integrity was also disrupted after exposure to UVB radiation (Fig. 2), especially with some longer exposure periods (150 s), perhaps due to changes in the phospholipid composition of the membrane or its membrane potential. This result also corroborates those obtained by Lu and Wu (2005b).

Another organelle affected by UVB radiation was the acrosome, a vesicular structure located in the anterior region of the sperm head that covers the nucleus. It is composed of a double membrane and contains proteolytic enzymes essential for sperm–oocyte fusion (Barth and Oko 1989; Flesch and Gadella 2000). Our results demonstrate that the extent of acrosomal damage increased with longer exposure times, with the exception of the 120 s group, which presented similar values to the 30 s group (Fig. 2). These alterations were detected immediately after UVB exposure and remained stable during cold storage for 24 and 48 h. Similar findings were reported by Seaver et al. (2009), who observed that spermatozoa with acrosomal damage could bind to the oocyte but were unable to penetrate it, thereby preventing fertilization. In the present study, UVB radiation exerted the most evident effects on membranous structures—especially the plasma and acrosomal membranes—due to their composition of phospholipids, proteins, and cholesterol. Although DNA integrity was evaluated due to its known susceptibility to UVB damage, no significant fragmentation was observed at the tested doses.Sperm motility is an important criterion for evaluating sperm quality (Au et al. 2002) as any failure in this process leads to loss during fertilization. Studies have used Computer-Assisted Semen Analysis to correlate sperm motility data with fertilization success. For example, improved fertility was associated with a decline in VSL in rats, an increase in linear motility in rainbow trout, and an increased in VAP in rooster, human, and ram sperm (Moore and Akhondi 1996; Lahnsteiner et al. 1998; Farrell et al. 1998; Youn et al. 2011; Guan et al. 2014). In general, sperm with high levels of VAP, VSL, VCL, LIN, and BCF show better migration and penetration of the cervical mucus (Mortimer 2000; Verstegen et al. 2002; Matos et al. 2008). In our data, VSL and VAP showed no significant changes with increase in exposure time (Table 1), or after 24 h of cold storage after UVB exposure (Table 2). In addition, ALH and STR (Table 1), LIN and STR (Table 2), BCF, DSL, LIN, STR, VSL, and WOB (Table 3) were higher after exposure. However, only further tests with artificial insemination can determine whether the observed changes in these kinematics indices tested actually lead to an increase or decrease in sheep fertility.

Motility is considered a good indicator of male fertility in several species (Gadea 2005; Kasimanickam et al. 2007; Broekhuijse et al. 2012), as well as for sheep (David et al. 2015; Yániz at al., 2015). In this study, progressive motility was not significantly different after any of the exposure periods or after cold storage. However, total motility was reduced at 30 s, 90 s and 120 s (Figue 1) of exposure to UVB radiation. The kinematics of sperm exposed to 150 s of UVB radiation (Table 1), were the same as the controls. Thus, the above results suggest that UVB irradiation required to stop gametic function is lower than that required to inhibit sperm motility. This means that while the irradiated sperm may be mobile, they may be functionally sterile (Seaver et al. 2009). The results reported here on the effects of UVB exposure on spermatozoa structures further confirm this inference.

Our findings support the hypothesis that mitochondria are among the primary targets of UVB radiation, showing greater sensitivity than nuclear DNA to UVB-induced oxidative stress. Similar to the early mitochondrial dysfunction described by Quadros et al. (2016) in Macrobrachium olfersi embryos—organisms naturally exposed to high solar irradiance in shallow, transparent waters—we observed damage consistent with cristae disruption, membrane integrity loss, and upregulation of mitochondrial fission proteins. Although our model involves mammalian gametes, the recurrence of mitochondrial damage as an early event suggests a conserved mechanism across taxa. In mammals, mitochondrial function is essential for sperm motility, capacitation, and successful fertilization, particularly during transit through the female reproductive tract. Therefore, acute mitochondrial impairment may compromise fertilization potential even in the absence of marked nuclear DNA damage. Although DNA is considered the primary chromophore for UVB radiation (Quadros et al. 2016), no significant DNA fragmentation was detected under the conditions tested in this study, which contrasts with previous reports describing immediate and severe genotoxic effects (Vetter et al. 1999; Browman et al. 2000; Hegedus et al. 2014).Some studies in mice have demonstrated that exposure to UVB radiation causes irreversible DNA damage in testicular cells, negatively affecting spermatogenesis (Shakyawaland Mahobiya, 2023). Additionally, some authors have indicated that increased ROS levels due to UVB radiation exposure lead to a decrease in membrane integrity a result of apoptosis and necrosis in spermatozoa. This demonstrated in mice exposed to UVB radiation, which resulted in reduced testicular weight and alterations in sperm biochemical parameters in mammals (Shakyawal and Mahobiya 2023). Other studies in humans have reported that UVB radiation exposure induces lipid peroxidation in sperm membranes, which correlates with reduced semen motility.

In conclusion, our findings demonstrate that low-dose UVB exposure induces structural damage to ram spermatozoa without compromising motility, and that subsequent cold storage at 5 °C does not exacerbate this damage. A results highlight that motility alone may not reflect the true fertilizing capacity of sperm, as cells with preserved movement can still exhibit significant subcellular impairments that may affect reproductive success.

## Data Availability

Not applicable.
